# The British Medical Association, Edinburgh

**Published:** 1898-07-30

**Authors:** 


### The British Medical Association, Edinburgh.

### THE ANNUAL GENERAL MEETING.

(From our Special Correspondent.)

This week Edinburgh is *en fête* to receive the British Medical Association. The citizens, medical and lay, have put forth every effort to welcome the members, and the hotel-keepers, with their usual consideration, have put up their prices to prevent overcrowding. A number of visitors made the journey by boat, and had a rather rough passage on Saturday and Sunday. On Monday the City began rapidly to fill, and long before the Reception Rooms at the Oddfellows' Hall were opened at four o'clock on that afternoon a goodly crowd had assembled at the doors. Within the hall, which was soon uncomfortably packed, members at once proceeded to register their names and to receive their membership cards and daily journal, together with tickets for the opening service in St. Giles' Cathedral and for the Provost's Reception. All other tickets had to be obtained at the opposite side of the room, and considerable annoyance was expressed in that tickets for a large number of the entertainments were not ready, so that members were compelled to make many fruitless journeys. On Monday evening there was the customary Presidential dinner party, and at half-past ten an enjoyable smoking concert was given by the Students' Union.

Tuesday morning was at first grey and dull, but at about ten o'clock the sun came out, and except for a low mist Edinburgh was soon looking its best. Leaving out of consideration a Council meeting, the principal feature of the morning was the inaugural service in St. Giles's Cathedral. This commenced at eleven, but two hours before that time a crowd of ladies was already besieging the western door. The service was most impressive, although the fact that most of the worshippers were in "tourist" costume detracted somewhat from the smartness of the congregation. The officiating clergy were the Very Rev. Dr. J. Cameron Lees, Dean of the Order of the Thistle and of the Chapel Royal, and Chaplain to the Queen; the Right Rev. Dr. Alexander Whyte, Moderator of the Free Church of Scotland; and the Right Rev. Dr. William Blair, Moderator of the United Presbyterian Church of Scotland. The inaugural discourse was finely delivered by Dr. Whyte, and a satisfactory collection was made in aid of the Royal Medical Benevolent College and the British Medical Benevolent Fund. In the afternoon the first general meeting was held, and Sir T. Grainger Stewart installed President in succession to Professor T. G. Roddick. After hearing the reports of various committees the meeting was adjourned till the evening. At half-past eight the M'Ewan Hall was crowded with members and ladies to hear the President's address, both large galleries being almost filled. The tedium of waiting was relieved by the fine playing of Mr. Collinson, organist to St. Mary's Cathedral. The arrival of the President was greeted with deafening applause, particularly from such parts of the hall as were occupied by students. Professor Grainger Stewart welcomed the Association to Edinburgh, and thanked the members for electing him to the presidential chair. His address, delivered with his customary charm of manner, was mainly of the "then and now" type, but his comparisons of the Old Royal Infirmary with the institution of the present day were fresh and instructive. After the customary votes of thanks a move was made to the Museum of Science, where the Lord Provost of Edinburgh held a successful, albeit somewhat overcrowded, reception. It must be admitted that the vast number of members and visitors is one of the most marked features of the meeting, and seems to have surpassed even the anticipations of the energetic local committee.

Wednesday morning was devoted to sectional work, most of the rooms being well filled, although there was a considerable proportion of the casual element, members strolling from section to section to foregather with each other, or to catch a glimpse of the notabilities who were taking part in the work. In the afternoon the address in Medicine was delivered by Professor T. R. Fraser. Later on the members adjourned from the M'Ewan Hall to Craig House, Morningside Drive, where a garden party was in progress, the guests being received by Sir Alexander Christisen and Dr. Clouston. The evening's entertainment consisted in a soirée by the local committee in the M'Ewan Hall and New University Quadrangle.

Thursday and Friday were to be signalised by addresses in surgery by Professor Annandale and in psychological medicine by Sir J. Batty Tuke respectively, while a large number of garden parties, receptions, and balls were promised. Saturday is, as usual to be devoted to excursions, some of which will reach as far as St. Andrew's, Perth and Dundee, Stirling and Bridge of Allan, and the Trossachs. On Friday an international golf match is likely to afford the Scottish members an opportunity of entertaining and instructing their visitors.

On the whole, the meeting appears to be, apart from its unavoidable unwieldiness, a social success. A pleasant feature is the number of distinguished foreign visitors, including Professors Gerhard, of Berlin; Osler, of Baltimore; Mikulicz, of Breslau; Sänger, of Leipzig; Kelly, of Baltimore; Dercum, of Philadelphia; Blanchard, of Paris; Von Ott, of St. Petersburg; Benedikt, of Vienna; Kocher, of Berne; Mosselli, of Genoa; Rosenstein, of Leyden; Snellen, of Utrecht; and Nuel, of Liège.

The Museums are both interesting, though the trade exhibitors at the Animal Museum complain that their stalls are not numbered, and that they have great difficulty in obtaining letters and packages. But the ordinary visitor recks not of such troubles; he is contented to admire the beautiful city in the blaze of the July sun, and to appreciate the courtesy and hospitality of the local members of the Association.

### THE ANTIDOTAL TREATMENT OF DISEASE.

Professor Fraser's Address.

The remarkable Address in Medicine delivered on Wednesday by Professor T. R. Fraser, at the Edinburgh meeting of the British Medical Association, is likely, for a time at least, to stand as a signpost for those who seek for antidotes by which to control the ravages of disease. When the Association last met in Edinburgh,

Dr. Warburton Begbie, that accomplished and universally-beloved physician, had in his address, after an elaborate survey of the history of medicine, arrived at the conclusion that no general doctrine had been propounded which satisfactorily explained the nature and production of disease, and that therapeutic advance had been obtained chiefly by adhesion to the classic method of rational empiricism. Now, however, the introduction of physical aids to our senses, and of chemical appliances and methods, has placed us in a new position, from which we have been able to make great advances in accuracy of diagnosis. But while the diagnosis of diseases has conspicuously advanced during the last quarter of a century, this advancement does not itself justify any claim to a nearer approach to the realisation of the highest aims and objects of medicine; for diagnosis has no immediate concern with the true nature or course of disease, and, until this has been determined, progress in treatment can only be tardy and unsatisfactory. As showing how diagnosis may reach a position of almost ideal perfection while treatment may remain in the initial stage of vague speculation, Professor Fraser instances the present state of our knowledge of nervous diseases. The solution of the complex problems in diagnosis presented by these diseases is undoubtedly a cause of satisfaction to the physician and frequently also to the patient. "To what extent, however, is the patient a gainer; to what extent is the object of diagnosis and of all medical knowledge fulfilled? It must be confessed that the gain in most cases is disappointing." In those cases, however, where it is possible to advance from diagnosis of the structural derangement to the determination of the actual cause of the disease, and when remedies are employed which have been proved to be curative as regards that cause, the disease, whatever be its position in the artificial nosology of nerve affections, may be arrested in its progress and even cured. The real remedy, in fact, has nothing to do with the particular disease, but is related only to its cause; and, as instances of this, syphilis, rheumatism, and malaria may be cited. In these the disease is not truly a product of the structural alterations which are present, but of a hurtful substance or poison capable, among other effects, of producing these structural alterations. Similar facts are observed with many ordinary poisons, and an association, highly significant in regard to the production of disease, is thus indicated, for these poisons produce changes which may be properly enough described as structural diseases. The important generalisation may then be made that the structural changes found in many diseases may be mere manifestations, associated with other effects of a cause which may be regarded as the essence of the disease, and that this essence is a toxic substance. Nor must we be content to confine our notions as to the toxic origin of disease to those ailments in which microbes have been discovered by which such toxins can be produced, or to those in which poisonous ptomaines have been swallowed, for the organism even in health is a veritable storehouse of toxic substances by which disturbances of function may be produced, and in such disturbances of function other poisons not found in the healthy body are generated, and may produce the symptoms of disease. The widely acting pathogenic influence of poisonous substances has, however, received its most definite and convincing support from the remarkable discoveries in bacteriology, for it has been proved that the pathogenic effects of the microbes of disease are mainly caused by the poisons which they produce. Thus, on every hand, facts accumulate to establish the doctrine of the toxic origin of disease, a doctrine which, it may be confidently anticipated, will have an endurance which former systems of medicine have not possessed.

It becomes, then, a matter of great interest to inquire what defence we can oppose to the disease-producing poisons by which we are constantly endangered, and on this subject much light is thrown by a study of the remarkable facts which have recently been discovered in connection with the poisons generated by pathogenic micro-organisms, and with other poisons of a very similar composition represented by the venom of serpents and by the vegetable products abrin and ricin, for it must be remembered that, in the known microbic diseases, neither the original disease nor the subsequently acquired protection is due to the microbe, but to the toxic substances produced by it. Thus a study of the manner in which protective substances, or anti-toxins, are produced will probably give the clue to much that is hopeful in therapeutics in other directions. By the results of such study a new era has been originated in practical medicine. A special fascination attaches itself to the subject of the antidotal treatment of disease, the newly-created serum therapeutics. Not only has it already obtained results which are of great value in regard to diphtheria, hydrophobia, and tetanus, but "the cure of pneumonia, of tubercle, of erysipelas, and of septicæmia is on the point of being realised . . . and the experimental data are being surely accumulated for completing the greatest triumph of preventive medicine by the discovery of an anti-toxic serum for the cure of small-pox." But that is not all; these are but the first fruits of the ample harvest that is ripening; in the meantime they are sufficient to supply materials for constructing the doctrine of the toxic origin of disease and of its antidotal treatment.

### THE PRESENT POSITION OF SURGERY.

### Professor Annandale's Address.

The address in surgery delivered by Professor Annandale on Thursday was devoted to a consideration of the Present Position of Surgery, and "for the sake of convenience, and partly also as is known to some here, that a discourse in this country is not considered complete unless delivered under from three to twelve heads," the subject was dealt with in (1) its scientific aspect; (2) its practical aspect; and (3) its moral aspect. The two first heads we will pass over, merely noting the admirable tone of common sense which pervaded all that they cover, and turn at once to Professor Annandale's remarks on the moral aspect of the surgery of to-day.

It was not of the practices of the so-called quacks or unqualified individuals that he wished to speak, but rather of practices inside the profession. It was sincerely to be regretted that in some quarters true, honest, and high feelings, the standard principles of our profession, were in the present day partially or wholly ignored, and that in consequence our profession was not always respected as it should be, and its members were looked upon by some as mere humbugs, in some cases not without reason, thinking more of fees and fee accumulation than of their patient's care or relief.

Three causes seemed responsible for this:—Active competition, untrained specialism, and society demands.

In regard to active competition, he did not think that this was so much due to the increase in the number of the medical men as to the fact that in many instances most of the remunerative work was done by the few, which left much hard work and poor pay for the remainder of the profession. As to specialism, he thought that honest specialism, such as was founded upon a thorough knowledge of all the different departments of the profession and upon an honest study of all the circumstances affecting the organ specialised, had added much to the practice of surgery. But specialism, if not practised under these conditions, even in the hands of qualified members of the profession was pure quackery, and quackery of the worst kind. There were few, he said, who had not met with cases in which mere symptoms had been treated by operation or otherwise, the real source of the diseased condition having been ignored, either through ignorance or for reasons which could only be classed as contemptible and degrading to the profession. A section of the public, or rather of that section of it which is termed "society," had done much to interfere with proper professional feeling. Men, women, and even young people read and discussed professional matters; books were published and advertised more to catch the eye of the public than to advance knowledge; and a certain class of newspapers and periodicals devoted one or more columns to professional subjects, and even gave gratuitous advice in the form of questions and answers. The manners, qualifications, and doings of the profession were freely discussed at afternoon teas, supposed failures were magnified, extravagant praise was bestowed, and wonderful surgical or other procedures were lauded, or even described with marvellous details added. And some there were, he feared, who took advantage of this extravagant popularity, and traded upon it in a manner which was not consistent with the high feelings which should influence all our professional relationships.

---

### AN EPISODE IN MEDICAL HISTORY.

### DR. BALFOUR'S ADDRESS.

The reminiscences of one who for fifty years or more has taken an active part in the teaching of his profession are sure to be interesting, and how interesting they may be was well shown by Dr. George W. Balfour in the address which he delivered on Wednesday at the opening of the section of medicine at Edinburgh. He took us back to the days when bleeding was the beginning and the end of all treatment in pneumonia, when one of the earliest lessons in surgery was to distinguish between an ordinary blood clot and one which was "buffed and cupped," and when blood letting was so thoroughly believed in as the panacea for every ailment that even if patients could be no longer safely bled they were certainly leeched and cupped. It may well then be believed how great was Dr. Balfour's astonishment in those days, on going to Vienna, to find the severest cases of pneumonia in Skoda's wards being treated with poultices, hay tea, and nothing else, unless much pain was complained of, when a few grains of Dover's powder were superadded. He had gone to Vienna to study homœopathy, which had just made a convert of one of our ablest professors, and it was, as he confesses, truly astonishing to behold "in bewildered amazement" a pneumonia melting away under the apparently magic influence of the decillionth of a grain of phosphorus. It was enough to convert at once to homœopathy anyone accustomed to the heroic methods which in those days were so commonly regarded as essential to the successful treatment of the disease. Perhaps, indeed, even so acute an observer as Balfour might have been led astray by such striking results, as they seemed, of homœopathic treatment had it not been for the fact that in Skoda's wards equally severe cases were being treated equally successfully by means of a little hay tea! As Dr. Balfour says, the first idea in the minds of those who observed the results of treatment by the infinitesimal doses prescribed by the homœopathists was that there must be some occult virtue developed by the various triturations and succussions, and some truth in the aphorism *similia similibus curantur.* Fortunately, the excellent results obtained by Skoda with his hay tea sufficed to dispel these clouds of mysticism, while the success of Dietl in the same class of cases with simple aqua colorata showed that there was nothing specific even in hay tea. Dr. Balfour has, we think, done well to recall this "almost forgotten episode in medical practice," because it is only by bearing in mind the ordinary conditions of medical practice at the time when homœopathy had its greatest vogue that we can understand the enthusiasm with which the new doctrine was then received. At the present time, when there is no professional bar and no professional prejudice against the use of any drug or any form of treatment by any physician, people not unnaturally wonder why certain men should profess to treat disease according to a particular system, each item of which is at the disposal of all the world. Fifty years ago, however, things were different. Homœopathy and the medicine of the day were distinct as the poles, not only, as they are now, in theory, but in actual daily practice, and the more firmly men believed, when they bled their patients, that they were curing diseases which, if let alone, would of necessity end fatally, the more were they in a manner bound to admit the mysterious influence of the triturations and infinitesimals under the use of which recovery also occurred. Treatment by hay tea, however, and by aqua colorata pricked the bubble. It was the dogmatic teaching that was wrong. When it was taught that "in the case of inflammation no one would think of trusting the safety of the patient to any other remedy than blood-letting" no one had an opportunity of seeing that this process, great as was the immediate relief which it often gave, was quite unnecessary, and it is but reasonable to believe, with Dr. Balfour, that, in the absence of the factitious contrast between the supposititious curative action of heroic medicine and that of infinitesimals, it is "unlikely that Hahnemann's wildly improbable ideas as to the preparations and powers of infinitesimals would have taken any hold upon the profession at all." It is by the wide diffusion of knowledge concerning the natural history of disease that the tyranny of dogmatism can be best prevented. Nor must we allow ourselves to believe that the days of dogmatism have passed away. Dr. Balfour is certainly in the right in the words with which he concludes his interesting address. "We are now on the threshold," he says, "of new discoveries, and of quite a new pathology, which is indeed but a higher development of ideas that have long been slumbering in the professional mind, the connecting links being Dwight, Raspail Hallier, and Pasteur, but which seem likely now to attain a development of the highest importance for the well-being of mankind. It is well, however, in the light of the past, to remember that disease may be recovered from under many different forms of treatment."

### MODERN OPERATING THEATRES.

Dr. Duncan's Address.

(For a Notice of this Address see page 313).

